# Joint AI-driven event prediction and longitudinal modeling in newly diagnosed and relapsed multiple myeloma

**DOI:** 10.1038/s41746-024-01189-3

**Published:** 2024-07-29

**Authors:** Zeshan Hussain, Edward De Brouwer, Rebecca Boiarsky, Sama Setty, Neeraj Gupta, Guohui Liu, Cong Li, Jaydeep Srimani, Jacob Zhang, Rich Labotka, David Sontag

**Affiliations:** 1 CSAIL, MIT, Cambridge, MA USA; 2 Harvard Medical School, Boston, MA USA; 3 Takeda LLC, Cambridge, MA USA

**Keywords:** Health care, Cancer, Computer science

## Abstract

Multiple myeloma management requires a balance between maximizing survival, minimizing adverse events to therapy, and monitoring disease progression. While previous work has proposed data-driven models for individual tasks, these approaches fail to provide a holistic view of a patient’s disease state, limiting their utility to assist physician decision-making. To address this limitation, we developed a transformer-based machine learning model that jointly (1) predicts progression-free survival (PFS), overall survival (OS), and adverse events (AE), (2) forecasts key disease biomarkers, and (3) assesses the effect of different treatment strategies, e.g., ixazomib, lenalidomide, dexamethasone (IRd) vs lenalidomide, dexamethasone (Rd). Using TOURMALINE trial data, we trained and internally validated our model on newly diagnosed myeloma patients (*N* = 703) and externally validated it on relapsed and refractory myeloma patients (*N* = 720). Our model achieved superior performance to a risk model based on the multiple myeloma international staging system (ISS) (*p* < 0.001, *Bonferroni corrected*) and comparable performance to survival models trained separately on each task, but unable to forecast biomarkers. Our approach outperformed state-of-the-art deep learning models, tailored towards forecasting, on predicting key disease biomarkers (*p* < 0.001, *Bonferroni corrected*). Finally, leveraging our model’s capacity to estimate individual-level treatment effects, we found that patients with IgA kappa myeloma appear to benefit the most from IRd. Our study suggests that a holistic assessment of a patient’s myeloma course is possible, potentially serving as the foundation for a personalized decision support system.

## Introduction

Multiple myeloma, the second most common blood cancer, has a global incidence of ~20,000 cases per year^1^. Clinical management of multiple myeloma patients is complex, both in terms of the factors that impact physician decision-making as well as the overall goals of care. Several factors, including the stage of the disease, transplant status, functional status, and genetic profile, are considered in the initial treatment decision^2,3^. Furthermore, effective care aims to maximize patient survival and time to disease progression while minimizing adverse events from therapy. When deciding on a treatment, striking an appropriate balance between optimizing patient survival while maintaining quality of life by limiting adverse events is intricate and requires frequent follow-up with patients. The International Myeloma Working Group recommends monthly (or bimonthly, depending on the therapy given as part of a follow-up or maintenance regimen) monitoring of patients receiving initial chemotherapy for response to treatment, disease complications, and toxic sequelae of therapy^1^.

However, despite prior work focusing on prediction of individual aspects of cancer management, such as overall survival (OS)^4–8^, progression free survival (PFS)^9^, adverse events (AEs)^10^, or biomarker forecasting^11^, no previous approach provides a holistic view of the patient’s disease course by modeling the different facets of multiple myeloma management simultaneously. This narrow focus limits the clinical applicability of such models.

**Fig. 1:**
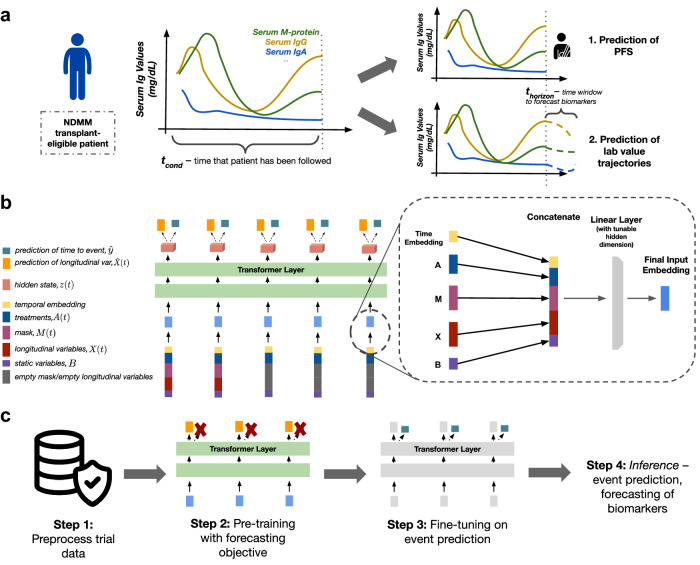
Problem setup and architecture of our model. **a** A schematic of a canonical scenario that our model might see during inference. We observe the clinical variables of a patient over an observation window of length *t*_cond_, at which point we are interested in predicting the survival outomes, potential adverse events, and evolution of relevant biomarkers. We denote the length of the time window over which we forecast the biomarkers, i.e., the forecasting window, as *t*_horizon_. The model architecture was evaluated over various combinations of *t*_cond_ and *t*_horizon_. **b** Diagram of the SCOPE architecture, indicating input variables, transformer layers, the number of which can be tuned, as well as the prediction heads. Additional architectural details can be found in the Methods section. **c** The training workflow consisted of two steps: pre-training and fine-tuning. We pre-trained the transformer model on the forecasting objective, and then fine-tuned on the event prediction task, keeping the rest of the model parameters frozen.

To address this gap, we developed a novel machine learning model called SCOPE, Simultaneous Cancer Outcome Prediction Estimator for multiple myeloma, which utilizes a transformer architecture^12^ and techniques from survival analysis. Transformer architectures are attention-based models that have emerged as a powerful approach for capturing long-range dependencies and modeling temporal relationships in sequential data. With their ability to dynamically weigh the importance of different elements in a sequence, attention-based models excel at capturing complex patterns and dependencies in longitudinal data. Adding a survival model as a prediction head affords us with a straightforward parametrization for predicting survival outcomes in the presence of censored data. SCOPE leverages all of these strengths. Our method jointly models the patient’s current and future disease course by (1) predicting survival outcomes such as progression-free survival PFS, OS, and AEs, (2) forecasting key disease biomarkers, and (3) estimating the effect of using different treatment strategies. A graphical depiction of the model architecture is presented in Fig. 1. Our model dynamically adapts to newly collected clinical data and performs the above tasks with observed clinical history of arbitrary lengths.

To train and evaluate our model, we used data from two randomized controlled TOURMALINE trials, MM1 and MM2. These randomized controlled trials (RCTs) investigated the effect of an ixazomib, lenalidomide, and dexamethasone (IRd) combination regimen on survival outcomes, compared to lenalidomide and dexamethasone (Rd). MM2, comprising newly diagnosed multiple myeloma patients (NDMM), was used for training and internal validation, while MM1, comprising relapsed and refractory multiple myeloma patients (RRMM), was used for external validation in a setting where the patient population was at a different stage in their disease process and was enrolled according to different inclusion criteria. Our joint modeling approach performed better than previous clinical baselines and compared favorably with highly specialized task-specific machine learning methods. All model code and algorithms are available at the following GitHub repository: https://github.com/clinicalml/SCOPE. The anonymized patient-level TOURMALINE trial data can be made available to investigators through the data-sharing portal: https://vivli.org/.

In addition to assessing predictive performance, we performed introspection of our model and demonstrated how our joint modeling approach automatically segments patients by myeloma subtype and reveals relationships between predicted biomarker trajectories and the risk of progression or adverse events.

Finally, the randomization of treatment assignment in both datasets provides a unique opportunity to reliably infer treatment effects from our model. Leveraging the treatment effect estimation capabilities of SCOPE, we showed that our method can be used as a tool to retrospectively identify patient subgroups that would benefit most from a particular treatment regimen. The versatility of our approach allows a clinician to understand a patient’s disease progression in response to a potential treatment from multiple viewpoints. Ultimately, this comprehensive clinical assessment can allow for more personalized treatment assignments and serve as a clinical simulator that supports discovery of biomarker associations as well as subgroups with heterogeneous treatment effects.

## Results

### Cohort statistics

Table 1 presents descriptive statistics for both MM1 and MM2. The MM2 cohort consists of 703 patients, while the MM1 cohort consists of 720 patients. The inclusion criteria were autologous stem cell transplant (ASCT) ineligibility as well as a new symptomatic myeloma diagnosis for MM2, whereas the inclusion criteria for MM1 included relapse from previously controlled disease. For each patient, we used a combination of baseline covariates (e.g., age, sex, etc.), longitudinal biomarkers (e.g., immunoglobulins, creatinine, etc.), and treatment as inputs. Details on the preprocessing of the data, including data normalization, imputation, sample splitting, and included variables are given in the Methods section. We performed a random 80/20 split of the data into a training set and test set. We further did five random 75/25 splits of the training set: each split consisted of a smaller training set that was used to train the model and a validation set that was used to tune the hyperparameters of the model, which can be found in the Methods section. All metrics are reported on the test set, averaged over the five trained models. The error bars in figures correspond to standard deviations, computed over the five model predictions. *p*-values were computed using a two-sample *t*-test for difference of means with *d*.*f*. = 4. We also reported evaluation metrics for patient subgroups stratified by the predominant heavy chain over-produced by plasma cells (IgG, IgA, light chain only, etc.). This stratification was chosen due to its clinical significance and its impact on current patient management. For instance, the heavy chain that is overproduced tends to be associated with variations in clinical presentation (e.g., IgD-myeloma usually presents with more aggressive disease and severe complications^13^), prognosis (e.g., IgA-myeloma is associated with worse prognostic outcomes compared to IgG-myeloma^14^), complications and clinical monitoring (e.g., tracking IgA versus IgG over time). Thus, understanding how SCOPE performs in these subgroups and validating that the trained model captures this structure is vital.

**Table 1:** Summary statistics of the MM1 and MM2 patient cohorts

Variable	MM2 Cohort	MM1 Cohort
Patients	703	720
Sex *n* (%)		
Female	351 (49.9%)	312 (43.3%)
Male	352 (50.1%)	408 (56.7%)
Race *n* (%)		
White	575 (81.8%)	614 (85.3%)
Asian	96 (13.7%)	64 (8.9%)
Native Hawaiian or other Pacific islander	1 (0.1%)	4 (0.6%)
Black or African American	23 (3.3%)	13 (1.8%)
American Indian or Alaska native	3 (0.4%)	1 (0.1%)
Other	5 (0.7%)	7 (1.0%)
Not reported	0 (0.0%)	17 (2.4%)
Ig Type *n* (%)		
IgG	403 (57.3%)	389 (54.0%)
IgA	142 (20.2%)	123 (17.1%)
IgD	10 (1.4%)	7 (1.0%)
IgE	3 (0.4%)	15 (2.1%)
IgM	3 (0.4%)	1 (0.1%)
Biclonal	23 (3.3%)	30 (4.2%)
No Heavy Chain	119 (16.9%)	155 (21.5%)
Median age [years] (min,max)	73 (48,90)	66 (30,91)
Median time from diagnosis [months] (min,max)	1.11 (0.3,52.9)	42.8 (3,306)
ISS Stage at study entry *n* (%)		
I	324 (46.1%)	458 (63.6%)
II	263 (37.4%)	176 (24.0%)
III	115 (16.4%)	86 (12.0%)
Actual Treatment *n* (%)		
Rd	349 (49.6%)	359 (49.9%)
IRd	354 (50.4%)	361 (50.1%)
Planned Treatment *n* (%)		
Rd	353 (50.2%)	362 (50.3%)
IRd	350 (49.8%)	358 (49.7%)

### Joint-modeling for multiple myeloma

Based on an available clinical trajectory of duration *t*_*cond*_ that consists of the baseline covariates, biomarker trajectory, and the past and planned treatments, SCOPE is able to jointly predict the risk of disease progression, the risk of death, the risk of a set of adverse events, and the future trajectories of biomarkers over a specified time horizon, *t*_*horizon*_. Each unit of time in our results is measured as a *treatment period*, corresponding to 28 days (~1 month).

**Fig. 2:**
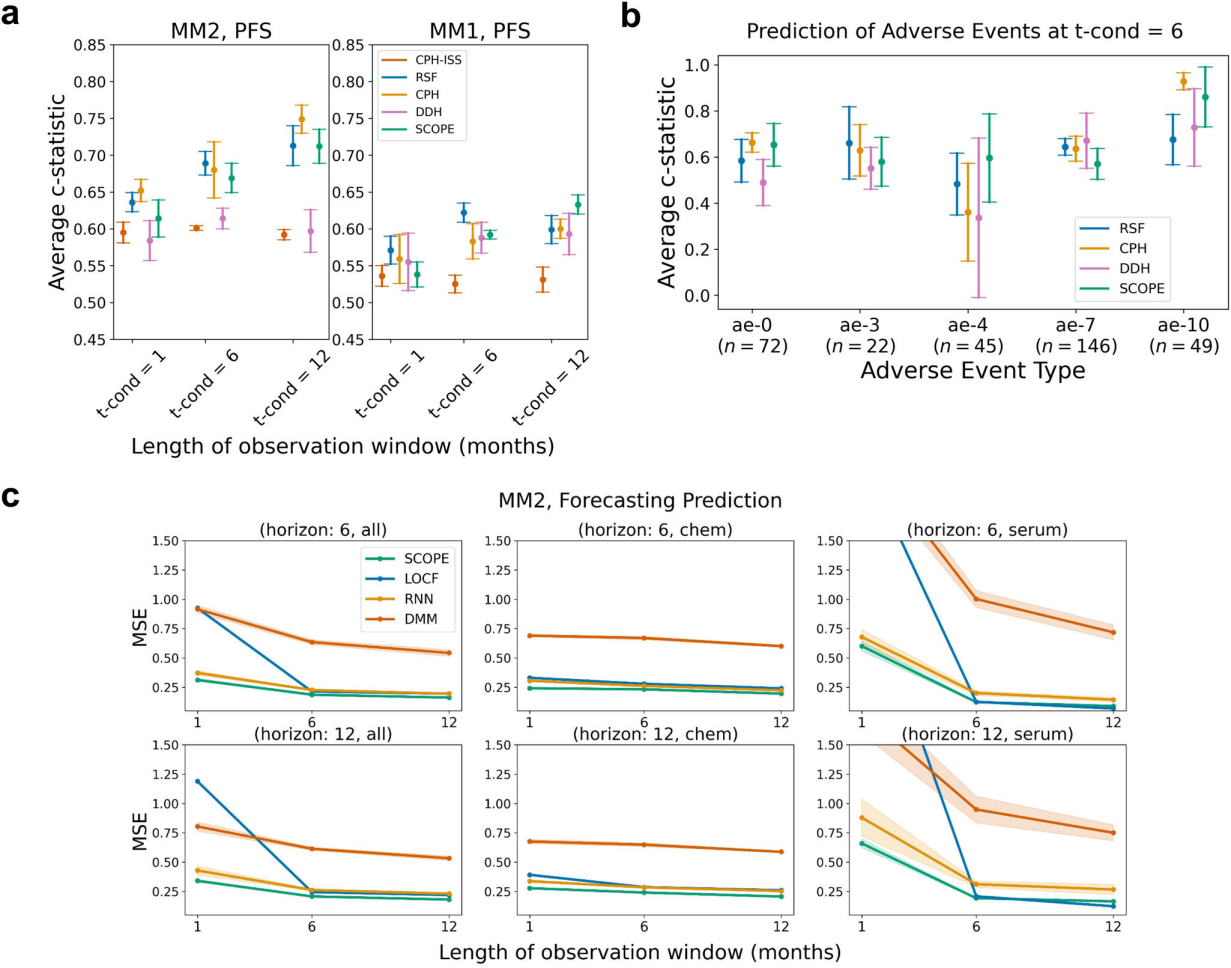
Event prediction and vitals forecasting performance evaluation. **a** *Event Prediction (Survival)*: We evaluated our model on two datasets: MM2, comprising NDMM patients, and MM1, comprising RRMM patients. We report concordance index based on inverse probability of censoring weights (C-index IPCW) averaged across three time quantiles (25th, 50th, and 75th quantiles) at different observation windows (1 month, 6 months, and 12 months). Looking at both MM2 and MM1 together, we found that the SCOPE had largely comparable performance to the RSF and CPH models, and significantly better performance than DDH and CPH-ISS (*p* < 0.001, Bonferroni corrected). We note the added benefit of having to train the SCOPE only once, compared to the two other model architectures, which required a separate model for each observation window and each event outcome. **b** *Event Prediction (AEs)*: We report average concordance index for multiple adverse events (filtering down to only ≥Grade 2 non-hematalogic events and ≥Grade 3 hematologic events) at the 6 months observation window (we refer to the Supplementary Material for results at other time points, Supplementary Fig. 2 and Supplementary Tables 1-11). The adverse events were mapped to shortened names as follows - ae-0: Acute Renal Failure, ae-1: Cardiac Arrhythmias, ae-2: Diarrhea, ae-3: Heart Failure, ae-4: Hypotension, ae-5: Liver Impairment, ae-6: Nausea, ae-7: Neutropenia, ae-8: Peripheral Neuropathies, ae-9: Rash, ae-10: Thrombocytopenia, and ae-11: Vomiting. We found that for those adverse events that were predictable from the data (i.e., hypotension, acute renal failure, neutropenia, and thrombocytopenia), SCOPE was competitive with highly-tuned, task-specific CPH and RSF models trained separately on each adverse event. **c** *Forecasting*: We plotted the mean squared error (MSE) of each model on forecasting different sets of variables, chemistry labs, serum immunoglobulins, and all lab values, over two forecasting horizons, 6 months and 12 months. Evaluation was done after having observed all of the patient’s data at three different time points (*t*_cond_): 1 month, 6 months, and 12 months. We found that SCOPE outperformed the other methods in all cases (*p* < 0.001, Bonferroni corrected). All error bars correspond to standard deviation, computed over the five model predictions.

Figure 2 shows a graphical representation of the results of different models on event prediction and biomarker forecasting. In terms of prediction of disease progression, we found that our model was competitive with other machine learning baselines. For the event prediction tasks (OS, PFS and AE), we compared our approach against a Cox proportional hazards model that uses the international staging system for multiple myeloma^15^ as its only covariate (CPH-ISS) and three machine learning models: a Cox proportional hazards models (CPH), a random survival forest (RSF), and a Dynamic-DeepHit model (DDH)^16^, each of which utilizes a larger set of baseline covariates and longitudinal biomarkers as input. Note that RSF and CPH can only use summary statistics of the clinical times series, while DDH can process the entire trajectory. These standard survival models have been widely used in a variety of risk prediction tasks across several chronic diseases^4,17–19^. CPH-ISS can be seen as the “standard of care” approach used by oncologists to stratify myeloma patients according to their risk, since it is based on a composite risk score, ISS, used in clinical practice to estimate patient survival. The different models were compared with respect to the concordance index for right-censored data based on inverse probability of censoring weights (C-index IPCW, note that a C-index of 0.5 corresponds to random guessing)^20^.

Importantly, SCOPE outperformed the CPH-ISS clinical baseline (*p* value < 0.0001). For example, on PFS for the MM2 study as shown in Fig. 2a, for *t*_cond_ = 6, we found a C-index IPCW of 0.67( ± 0.02) for SCOPE, 0.60( ± 0.00) for CPH-ISS, 0.61( ± 0.01) for DDH, 0.68( ± 0.04) for the Cox model, and 0.69( ± 0.02) for RSF. We note that this trend persisted across patient subgroups with a different dominant immunoglobulin heavy chain (more results are provided in the Supplementary Material, Supplementary Tables 1–3). For OS prediction, we observed a similar result, where SCOPE outperformed CPH-ISS, except for the baseline time point.

For biomarker forecasting, we found that SCOPE outperformed all other methods (*p* value < 0.0001, with Bonferroni correction). We compared SCOPE against a recurrent neural network (RNN) and a deep Markov model (DMM), two state-of-the-art time series forecasting models, and a last observation carried forward mechanism (LOCF). We used the mean squared error (MSE) over the forecasting horizon as our evaluation metric. As shown in Fig. 2c, for an observation window of 1 month (i.e., conditioning on all the patient’s data at 1 month) (*t*_cond_ = 1) and a horizon of 6 months (*t*_horizon_ = 6), there was an MSE of 0.31( ± 0.01) for SCOPE, 0.37( ± 0.02) for the RNN, 0.92( ± 0.03) for the DMM, and 0.93( ± 0.0) for LOCF. We note that the performance gap decreases with an increasing length of the observation window, due to the decreasing variability of the clinical trajectories over time (as shown in the Supplementary Material, Supplementary Fig. 3).

In addition to forecasting clinical trajectories and predicting disease progression and overall survival, SCOPE is able to predict the onset of particular adverse events. For instance, as shown in Fig. 2b, we found that our model could predict the occurrence of acute renal failure (C-index IPCW 0.62( ± 0.07)), hypotension 0.66( ± 0.14), neutropenia 0.59( ± 0.07), and thrombocytopenia 0.85( ± 0.10). Results for all remaining observation windows and patient subgroups are available in the Supplementary Material (Supplementary Fig. 2, Supplementary Tables 1–11).

### SCOPE introspection

We conducted analyses to interpret the learned hidden patient representations in SCOPE. In principle, these correspond to the hidden states of the last transformer layer at different time steps. We investigated which dimensions of the hidden state in the transformer layer were associated with a higher event/progression risk and how this related to trends in the biomarker predictions. This was done for two event-biomarker pairs: progression with serum M-protein and hemoglobin as well as acute renal failure with creatinine. For each dimension of the hidden state, and for each time step, we computed the Pearson correlation (1) between the value of the hidden state at said dimension and the predicted risk of progression (or acute renal failure), and (2) between the value of the hidden state at said dimension and the predicted value at the next time step for the biomarkers of interest. All analyses were performed with a single model trained on the MM2 cohort.

**Fig. 3:**
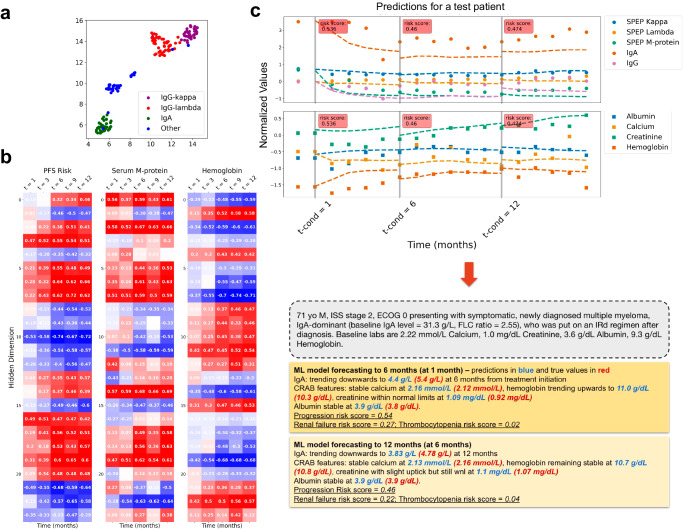
Model introspection and predictions visualization. **a** We computed a UMAP embedding of the transformer hidden state at the first time point and then colored each patient by their myeloma subtype. We found that the hidden state captured the underlying myeloma subtype structure, including subtypes delineated by which heavy and light chains dominated the disease process. **b** At five different time points, we computed the correlation between the hidden state for all patients and the risk of progression (*predicted value*), serum M-protein level (*feature*), and hemoglobin level (*feature*), respectively. The number of hidden dimensions is 64, but only the dimensions that had at least one time point above 0.4 were shown. Red indicates a positive association between the hidden state value and the feature or prediction, whereas blue indicates a negative association. We saw that in dimensions where there was a growing risk of progression, a sensible change in the forecasts was noted, i.e., serum M-protein level tended to go up, and hemoglobin tended to go down, indicating anemia. **c** We generated samples of several biomarkers, including immunoglobulins and chemistry labs, from the model at three different conditional time points (one month, six months, and twelve months) for a test patient. In the sample plots, the solid dots denote the ground truth values, and the dotted lines are the predictions. At each time point, we also report a risk score for disease progression, defined in the “SCOPE Introspection” section under Methods. These predictions enable a clinical assessment of individual patients that can be summarized into a clinical vignette for the physician. In this vignette, the predictions are in blue and the true values are in red. Details about biomarker normalization are available in the Methods section.

To interpret the learned hidden states of the last transformer layer in SCOPE, in Fig. 3a, we show a UMAP visualization of the hidden state vector at baseline for each patient in the test set. We found that the hidden state captured myeloma subtypes determined by the involved heavy chain and light chain. Although this result does not directly imply good predictive performance, it points to a meaningful patient representation.

The correlations between the hidden states and risk of progression, serum M-protein, and hemoglobin are presented in Fig. 3b for five observation windows (one, three, six, nine, and twelve months). Each cell is colored according to the correlation strength. For clarity, we removed the hidden dimensions that had low correlation (below a threshold of 0.4). We found that for hidden dimensions indicating a higher risk of progression, sensible correlations with M-protein and hemoglobin predictions were observed. Namely, serum M-protein level was predicted to go up, given the hidden state as input, and hemoglobin was predicted to go down, indicating anemia, which agrees with basic clinical reasoning. We give another example of sensible associations between GFR and acute renal failure in the Supplementary Material (Supplementary Fig. 1). This demonstrates the ability of our model to uncover associations between longitudinal biomarkers and clinical events.

Finally, we demonstrate the granularity of the model’s predictive capabilities by reporting the predictions for disease progression and forecasts for an individual patient. In Fig. 3, we show model predictions of disease progression and forecasts of several biomarkers over multiple horizons and observation windows for an IgA-dominant myeloma patient from the test set. We found that our model generally predicted the correct trends for both immunoglobulins, e.g., downtrend in IgA in response to treatment, and biomarkers related to CRAB (Hypercalcemia, Renal failure, Anemia, and Bone lesions) complications (e.g., calcium, hemoglobin, and creatinine). We used these predictions to create a more meaningful clinical summary of the patient’s disease course, as shown in Fig. 3c.

### Treatment effect estimation and discovering heterogeneous subgroups

**Fig. 4:**
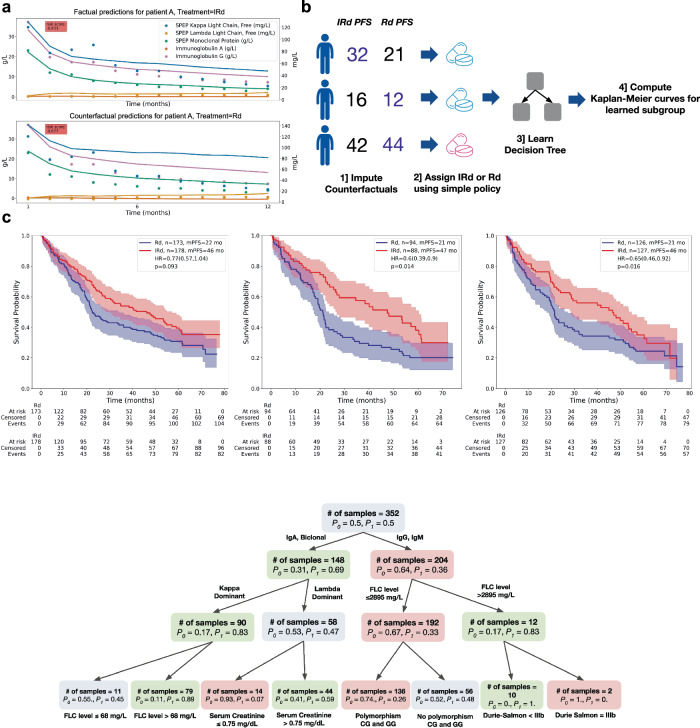
Treatment effect estimation and discovering heterogeneous subgroups. **a** We plot the predictions from baseline of a single patient’s serum immunoglobulins over both the factual treatment (here IRd - top) and the counterfactual treatment (Rd - bottom), demonstrating SCOPE's ability to model counterfactuals. Dots represent the observed values, and solid lines represent the predictions of SCOPE from baseline. The variables with units g/L are measured along the right axis (SPEP Kappa, Lambda, and Monoclonal Protein), and variables with units mg/L are measured along the left axis (IgA and IgG). **b** Diagram of a proof-of-concept subgroup discovery analysis. **c** On the left, we show the Kaplan-Meier curves for the original treatment and control groups in a left-out set of MM2 patients. In the middle, we show the Kaplan-Meier curves for the treatment and control groups in a learned subgroup of MM2 that suggest greater differential survival between IRd and Rd for this patient subgroup. On the right, we show the Kaplan-Meier curves for the interpretable subgroup learned to replicate the subgroup found with our model. The *p*-values were computed with a log-rank test. **d** Visualization of the learned decision tree, which was trained using the subgroup assignments as labels and patient baseline data as features. Each node contains the number of patients, and the proportion of the samples who are labeled as getting Rd (*P*_0_) or IRd (*P*_1_), respectively. A node is colored red if *P*_0_ > *P*_1_, green if *P*_0_ < *P*_1_, and blue if *P*_0_ ≈ *P*_1_.

The predictions of SCOPE use the past and planned treatment assignments. Because the MM2 dataset was collected in the context of a randomized clinical trial, we also used our model to investigate the impact of *counterfactual* treatments on individual patients (note that a counterfactual treatment is an alternative treatment strategy than the one actually received by the patient; a counterfactual prediction projects what would have happened if the patient had received another treatment). We show an example of forecasting progression risk and biomarker trajectories under planned and counterfactual treatment assignments for a test patient in Fig. 4. For this patient, SCOPE assigns a lower risk score when treated with IRd than with Rd. Projections with Rd predict a marked increase in serum kappa light chain, serum M-protein, and IgG over time compared to a scenario where the patient would be treated with IRd.

As our model generates individual predictions with different treatment assignments, it also enables the discovery of sub-populations with heterogeneous treatment effects. To this end, we computed individual treatment effects on disease progression risk for each patient and identified the subgroup that benefits most from the treatment using a simple policy learned from MM2 training data. To enhance interpretability, we used a shallow decision tree to visualize the identified subgroup.

Following the original trial analysis^21^, we performed a stratified Cox regression on a held-out set of MM2 patients before doing subgroup discovery. This analysis resulted in a barely non-significant treatment effect (*N* = 351, *p* = 0.093), which was similar to the significance level obtained on the whole MM2 cohort (*N* = 703, *p* = 0.073). In contrast, in the subgroup identified with our approach, the effect of treatment was significant (*N* = 182, *p* = 0.014), using the same stratified Cox regression analysis. A shallow decision tree trained to replicate the subgroup assignment strategy of our model resulted in a subgroup with similar significance with respect to treatment efficacy (*N* = 153, *p* = 0.016). The inclusion criteria for the discovered subgroup, as reflected by the learned decision tree, are presented in Fig. 4. The relevant clinical variables are the multiple myeloma subtype, free light chain concentration, serum creatinine, CG and GG polymorphisms, and the Durie-Salmon stage. Notably, the learned subgroup suggests that IgA patients are more likely to benefit from IRd than IgG and IgM patients. Details on counterfactual predictions are given in the Methods section.

### Generalizability to relapsed and refractory multiple myeloma patients

We evaluated our model on the MM1 trial data, which consisted of patients with relapsed myeloma who had received prior lines of therapy. The evaluation of our model on an external cohort with different disease characteristics compared to the training population served as external validation and measured the model’s ability to generalize. In the PFS prediction task (Fig. 2a), we observed a decrease in performance across all models, as expected due to the change in cohort characteristics. However, SCOPE had the lowest average decrease in concordance index (0.077) compared to RSF (0.083) and CPH (0.11). DDH had a lower decrease and CPH-ISS had a comparable decrease (0.076 vs 0.077) to SCOPE, but with poor absolute performance. Additionally, we found that our model outperformed all other models in forecasting performance on the MM1 cohort (see Supplementary Material, Supplementary Table 11), despite a slight increase in mean squared error across each *t*_cond_.

## Discussion

To our knowledge, this study is the first to develop and evaluate a holistic attention-based architecture for multiple myeloma management: one that jointly forecasts core myeloma biomarkers and predicts risk of important clinical events. Managing multiple myeloma involves a crucial balance between maximizing survival, minimizing adverse events, and keeping track of core biomarkers, which serve as a sufficient proxy for disease burden, over time^3^. These principles hold true in a wide range of malignancies and inform clinical management more generally^22–24^. Our model reflects these clinical management objectives by jointly modeling time to event and longitudinal biomarkers.

Joint modeling of time to event and longitudinal data has a long history in the biostatistics literature, where it has been used to improve the efficiency of estimators^25^. Most approaches for joint modeling have relied on linear mixed-effect models and classical survival models, such as Cox regression and Accelerated Failure Time models^26,27^. For example, Proust-Lima et al. developed and validated a dynamic prognostic tool for prostate cancer recurrence using repeated measures of post-treatment prostate-specific antigen^28^. However, these prior approaches largely focus on a single event and univariate biomarker predictions, in contrast to our work, where we consider multivariate forecasting and prediction of multiple events. In general, previous literature has predominantly employed less expressive models, such as linear models, in contrast to the transformer architecture that underlies our model. While recent work has leveraged transformers for tasks involving temporal data^29,30^, we are not aware of implementations for joint modeling of clinical data.

On the joint modeling tasks, our experiments showed that SCOPE outperforms other state-of-the-art longitudinal models for biomarker forecasting, over all observation windows and prediction horizons. Additionally, our model was comparable to tailored event prediction models, and superior to CPH-ISS, both in terms of prediction of disease progression and overall survival. Unlike these approaches, our model can do inference given data over any observation window. We further showed that our model can accurately predict the occurrence of serious adverse events such as heart failure, hypotension, acute renal failure, neutropenia, and thrombocytopenia. The ability of our model to jointly predict these aspects of the disease provides clinicians with a more holistic and personalized picture of a patient’s disease progression. In particular, we demonstrated in our introspection experiments how the predictions of our model for an individual patient can be summarized into a concise, tailored clinical assessment over time.

A limitation to the deployment of machine learning models in clinical practice is their black-box nature. We performed an in-depth investigation of the model by studying the patient representations learned by our model and assessed their clinical relevance. We showed that the learned hidden states represented the patient’s underlying disease state and progression in two ways. Firstly, the hidden representations tended to cluster by myeloma subtype, which is associated with differences in prognosis and severity of disease^13,14^. Secondly, the model recovered clinically sensible correlations (e.g., higher serum M-protein being positively correlated with higher risk of disease progression). This introspection can also potentially serve as the basis for discovery of novel associations, which is especially pertinent in the case of genomic marker discovery.

We established the potential of SCOPE as a response-surface model with which we can estimate counterfactual outcomes and thereby estimate personalized treatment effects, i.e., in this case, the effect of IRd compared to Rd for individual patients. Providing counterfactual predictions can both help clinicians make more informed treatment decisions and serve as a tool for uncovering heterogeneous patient subgroups. As a demonstration of the latter, we proposed an algorithm that uncovers a patient subgroup, i.e., IgA and biclonal myeloma patients with high baseline free-light chain levels, for whom IRd leads to statistically significantly better PFS than Rd. In the original pre-specified subgroup analysis of the TOURMALINE-MM2 trial^21^, median PFS was statistically significantly higher for patients who had certain high-risk cytogenetics, which included del(17p), *t*(4;14), *t*(14;16), and amp(1q21) abnormalities, as well as patients who had low creatinine clearance (≤ 60 mL/min). Subgroups based on the nature of the monoclonal protein (e.g., IgA-dominant, IgG-dominant, etc.) were not assessed. However, we also find, in our learned decision tree, that patients with high serum creatinine, due to potentially low creatinine clearance, were more likely to benefit from the IRd regimen. We emphasize that this proof-of-concept analysis is hypothesis-generating and that further work is necessary to validate this result.

We focused on two treatment regimens, IRd and Rd, to streamline model development and due to the availability of high-quality data from the TOURMALINE trials. However, as we aim to move towards clinical deployment of our models, the training data can be expanded to include the diverse set of treatment regimens available for multiple myeloma. For example, including patients in the training set who received different variants of induction therapies, e.g., triplet therapy followed by ASCT or extended daratumumab-based quadruplet therapy in a transplant-ineligible patient, would allow for the model to be applicable to more patient populations and to assess additional counterfactuals. As more data is collected, periodic retraining of our model may be necessary to handle inference under other treatment policies.

Past work in the treatment effect estimation literature has extensively explored using various models, including Bayesian regression trees and neural networks, as response-surface models, to estimate the expected outcome of interest conditioned on a treatment and patient covariates^31–33^. We see our approach as an important step towards using transformer-based models for heterogeneous treatment effect estimation in *real-world data*.

Data driven models are by definition highly dependent on the input data they were trained on. This can lead to poor performance when these models are evaluated on a patient cohort with different characteristics. To quantify the generalization ability of our approach, we evaluated our model on an external patient cohort (MM1) consisting of relapsed and refractory multiple myeloma patients. While SCOPE did incur an expected loss of performance, our results suggest our model is more robust than other state-of-the-art baseline models. Still, caution should be exercised when using our approach (or any other approach) on other patient cohorts not included in the training data.

The design of our model was motivated by providing clinicians with the most holistic and personalized view of the disease state of multiple myeloma patients. Our model represents a step in that direction, though many hurdles towards clinical deployment remain. While we attempted to mitigate and quantify these limitations, prospective evaluation of the model in a clinical trial will be crucial for an eventual translation to clinical practice.

## Methods

### Data pre-processing

#### Sample splitting

On the MM2 data, we performed a random 80/20 split of the data into training and test sets. We created five additional random 75/25 splits of the training set into a smaller training set that was used for model training and a validation set used for hyperparameter tuning. For the generalizability experiments, we evaluated the model on all MM1 patients.

#### Included variables

The data available for each patient includes baseline variables that do not evolve over time (*B*), longitudinal variables (*X*), and treatment variables (*A*). We included the following variables for our analysis, filtering out any variable that were below a missingness threshold of 15% for the baseline variables and 70% (computed over the entire follow-up time) for the longitudinal variables.

##### Baseline variables, *B*

- Age, Race, Sex
- Time since diagnosis (months)
- Plasma cell (%), Bone marrow plasma cells (%)
- Baseline involved free light chain level (mg/L), Baseline creatinine (mg/dL), Baseline creatinine clearance (mL/min), Baseline albumin (g/dL)
- Presence of polymorphisms (Separate binary covariates for presence of CC, CG, GG specific changes are included as well).
- Cytogenetics result (abnormal, normal, or indeterminate)
- Baseline plasmacytoma observed
- History of bone lesions
- Extramedullary disease at study entry
- Measurable disease flag (Serum M-protein, Urine M-protein, Both SPEP and UPEP, Serum FLC)
- Liver function based on baseline tests
- ISS at baseline
- Myeloma type
- L-chain type at baseline (kappa, lambda, biclonal)
- Durie-Salmon Stage at initial diagnosis
- Lytic bone disease at initial diagnosis
- ISS stage at initial diagnosis
- ISS stage at study entry
- ECOG
- Beta-2 microglobulin (categorical)
- Free light chain ratio (categorical)
- Cytogenetics abnormality (categorical, high risk vs standard)
- Skeletal survey done at baseline
- Lytic bone lesion present at baseline
- del17 present
- t(4;14) present alone
- 1q amplification status
- Treatment arm

##### Longitudinal variables, *X*

- Albumin (g/L)
- Alkaline Phosphatase (U/L)
- Alanine Aminotransferase (U/L), Aspartate Aminotransferase (U/L), Bilirubin (umol/L), Blood Urea Nitrogen (mmol/L)
- Calcium (mmol/L), Chloride (mmol/L), Corrected Calcium (mmol/L), Glucose (mmol/L), Potassium (mmol/L), Magnesium (mmol/L), Phosphate (mmol/L)
- Carbon Dioxide (mmol/L)
- Creatinine (umol/L), Glomerular Filtration Rate Adj for BSA via CKD-EPI (mL/min/1.73m2)
- Hematocrit, Hemoglobin (g/L), Lymphocytes (10^9^/L), Monocytes (10^9^/L), Neutrophils (10^9^/L), Platelets (10^9^/L), Leukocytes (10^9^/L)
- Lactate Dehydrogenase (U/L)
- Protein (g/L), Serum Globulin (g/L), Sodium (mmol/L), SPEP Gamma Globulin (g/L), Free SPEP Kappa Light Chain (mg/L), Free SPEP Kappa Lt Chain/Free Lambda Lt Chain, Free SPEP Lambda Light Chain (mg/L), SPEP Monoclonal Protein (g/L), Serum TM Albumin/Globulin, Immunoglobulin A (g/L), Immunoglobulin G (g/L), Immunoglobulin M (g/L)
- Urine Albumin (%), UPEP Monoclonal Protein (mg/day), Urate (umol/L)

##### Treatment variables, *A*

Dosages of lenalidomide, ixazomib, and dexamethasone were included in the longitudinal treatment information if the patient was assigned to the treatment group. Otherwise, if the patient was assigned to the control group, dosages of lenalidomide, dexamethasone, and a placebo pill were given.

#### Normalization

Our model relies on a wide range of clinical and biological signals that have different scales and variability. We normalized the baseline variables, *B*, by subtracting the mean of each variable and scaling by the inverse of the standard deviation. For each fold, the mean and standard deviations were computed on the training set alone.

For the longitudinal variables, *X*, we used a normalization strategy aimed at being clinically informative while still enforcing an informative variance over time. Immunoglobulin markers tend to be very large at baseline and quickly decrease over time. A typical standard scaling of this variable would then tend to mask the variability in the immunoglobulin levels after the first decrease. Yet, to predict progression, the ability to read the future trajectories of immunoglobulins is crucial.

Our normalization strategy used normal clinical ranges for each variable. We first normalized by the normal range. Letting (*α*_*j*_, *β*_*j*_) be the lower and upper bounds of the normal range of a variable *j*, we first computed

1 $${X}^{j,* }=\frac{4* ({X}^{j}-{\alpha }_{j})}{{\beta }_{j}-{\alpha }_{j}}-2$$

For SPEP light chains, UPEP proteins, and urate, we further scaled the variable by a factor $\frac{1}{5}$, to accommodate their values being significantly higher than the normal range. We then incorporated an invertible non-linearity to retain variability of the signal in the lower ranges. The final normalization for a longitudinal variable *j* was obtained using the following formula,

2 $${X}^{j,{\dagger} }=\frac{7}{1+{e}^{-0.25\cdot {X}^{j,* }}}-3.5.$$

#### Imputation

For imputation of missing baseline features (i.e., *B*), we distinguished between categorical features and continuous-valued features. For the categorical features, we imputed the missing values with the most frequent value across all patients. For the continuous features, we mean imputed the missing values. For imputation of lab features (i.e., *X*), we mean imputed the lab features at the baseline time point (*t* = 0), and then forward filled the rest of the missing values over time, if any.

### SCOPE Architecture

The architecture in our experiments was built around a transformer encoder with continuous temporal embeddings and coupled with Cox proportional hazards (CPH) prediction heads. Transformer architectures^12^ are attention-based models that can capture long-range dependencies in sequential data. The CPH prediction head affords a straightforward parameterization for predicting survival outcomes in the presence of censored data. SCOPE leverages all of these strengths. A graphical depiction of the architecture is presented in Fig. 1a.

Notationally, at time *t*, *X*(*t*) denotes the longitudinal covariates, *M*(*t*) denotes the mask of observed longitudinal covariates (note *M*(*t*) = 1 indicates the value is observed and *M*(*t*) = 0 indicates the value is missing), and *A*(*t*) denotes the treatments administered as well their dosages. *B* denotes baseline covariates that we assume do not change over time. Based on an available clinical trajectory of duration *t*_*cond*_ that consists of the baseline covariates, *B*, the biomarker trajectory, {*X*(*t*), *M*(*t*) : 0 ≤ *t* < *t*_*cond*_}, and the past and planned treatments {*A*(*t*) : 0 ≤ *t*}, SCOPE is able to jointly predict the risk of disease progression $({\hat{y}}_{PFS})$, the risk of death $({\hat{y}}_{OS})$, the risk of adverse events $({\hat{y}}_{AE})$, and the future trajectories of biomarkers over a specified time horizon, *t*_*horizon*_. Each unit of time in our results is measured as a *treatment period*, corresponding to 28 days (~1 month).

**Fig. 5:**
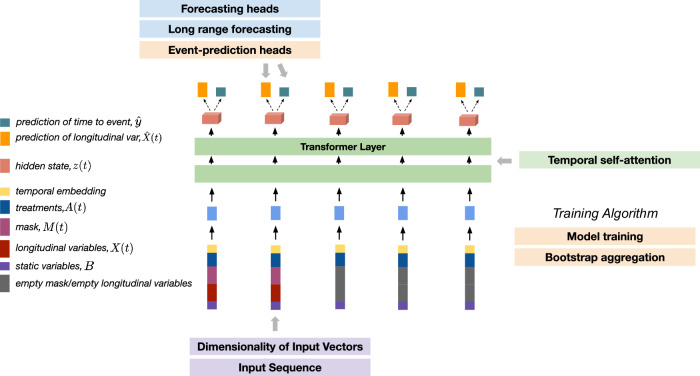
Organization of the methods section with respect to the SCOPE architecture. The first two sections reflect details about the input data. The next section gives the mathematical details for temporal self-attention, which is the workhorse for each transformer layer. Then, there are three sections on how the prediction heads, both forecasting and event prediction, are parameterized. Finally, there are two sections on the training algorithm.

Conceptually, our transformer-based model encodes the sequence of clinical observations into a sequence of hidden states that capture the relevant information in the clinical trajectories. These hidden states are shared jointly across all prediction tasks. A different prediction head, parameterized by a neural network, is learned for each individual task. For further architectural and optimization details, see below. Each section is organized according to architectural element and shown in Fig. 5 for clarity.

#### Dimensionality of input vectors

The dimensions of each vector are as follows: $X(t)\in {{\mathbb{R}}}^{N\times {D}_{{{{\rm{lab}}}}}}$, $M(t)\in {{\mathbb{R}}}^{N\times {D}_{{{{\rm{lab}}}}}}$, $A(t)\in {{\mathbb{R}}}^{N\times {D}_{{{{\rm{treat}}}}}}$, and $B\in {{\mathbb{R}}}^{N\times {D}_{{{{\rm{base}}}}}}$, where *N* is the number of patients in the dataset, *D*_lab_ is the number of lab covariates, *D*_treat_ is the number of treatment covariates (e.g., dosage of each drug), and *D*_base_ is the number of baseline covariates. We let ${T}_{\max }$ be the maximum follow-up time for any patient, such that $t\le {T}_{\max }$.

#### Input sequence

Let *E*(*t*) be a *τ*-dimensional time embedding of the following form: ${E}_{2k}(t):= \sin \left(\frac{t}{{T}_{\max }^{2k/\tau }}\right)$, ${E}_{2k+1}(t):= \cos \left(\frac{t}{{T}_{\max }^{(2k+1)/\tau }}\right)$, where 0 ≤ *k* ≤ *τ*/2 − 1 indexes the *E*(*t*) vector. Then, the input embedding to the SCOPE is simply a concatenation of [*B*, *X*(*t*), *M*(*t*), *A*(*t*), *E*(*t*)] taken through a linear layer. Both the time embedding dimension, *τ*, and the input embedding dimension were tuned on the validation set. See Table 2 for a full set of hyperparameters and what values we search over for each.

**Table 2:** Hyperparameters used for training SCOPE

Name	Description	Values
Hidden dimension (*D*_*z*_)	Dimension of the hidden vectors in the transformer layers.	[16, 32]
Dropout	Probability of dropout in the transformer layers.	[0.1, 0.2]
Rollout-window (*K*)	Number of future hidden used as inputs to the event prediction modules.	[0, 1]
Number of layers	Number of transformer layers in the architecture.	2
Non-linearity	Whether to use a linear or non-linear prediction function for the event prediction.	[True, False]

#### Temporal self-attention

We refer to ${z}_{t}^{(0)}=emb([B,X(t),M(t),A(t),E(t)])$ as the input embedding of our model for the observations at time *t*. The transformer encoder layer performs the following computation. For a temporal embedding at layer *l*, ${z}_{t}^{(l)}$, we compute query (*q*), key (*k*), and value (*v*) vectors as

3 $${q}_{t}^{(l)}={W}_{q}{z}_{t}^{(l)}$$

4 $${k}_{t}^{(l)}={W}_{k}{z}_{t}^{(l)}$$

5 $${v}_{t}^{(l)}={W}_{v}{z}_{t}^{(l)}$$

These vectors are then concatenated into their respective matrices *Q*^(*l*)^, *K*^(*l*)^, and *V*^(*l*)^. The output embedding for time *t*, ${z}_{t}^{(l+1)}$, is given by

6 $${z}_{t}^{(l+1)}={f}_{l}\left({{{\rm{softmax}}}}\left(\frac{{q}_{t}^{(l)}{({K}^{(l)})}^{T}}{\sqrt{{d}_{k}}}\right){V}^{(l)}\right),$$

where *f*(⋅) is a multi-layer perceptron (MLP), and *d*_*K*_ is the dimension of the input embedding vector. In the next few sections, we formalize how to do forecasting and event prediction via separate “prediction heads”.

#### Forecasting heads

We perform forecasting based on the sequence of embeddings after the last transformer layer, ${z}_{t}^{(L)}$. In our experiments, we set *L* = 2. We use *D*_*z*_ to refer to the dimension of the hidden vectors. We predict the future values of biomarkers by using a dedicated MLP (*f*_*pred*_(⋅)) with the last embeddings as input:

7 $${\widehat{X}}_{t+1}={f}_{{{{\rm{pred}}}}}({z}_{t}^{(L)})$$

#### Long range forecasting

The forecasting prediction heads predict the next values of biomarkers conditioned on the clinical trajectory until time *t*. To predict over horizons longer than a single step ahead, we re-use the prediction at the previous time step in the next input vector. Writing ${z}_{t}^{(L)}(B,X(t),A(t),E(t))$ as the output embedding for time *t*, where we make the dependence on the inputs explicit, we compute the next embedding as follows,

8 $${z}_{t+1}^{(L)}={z}_{t+1}^{(L)}(B,{\widehat{X}}_{t+1},A(t),E(t))$$

9 $${\widehat{X}}_{t+2}={f_{{{{\rm{pred}}}}}}({z}_{t+1}^{(L)})$$

where ${\hat{X}}_{t+1}$ is obtained with Eq. (7). This procedure can be repeated to produce forecasts over arbitrary prediction horizons. The forecasts therefore only depend on *X* until time *t*. We still use the future treatment assignments, which allow one to generate the trajectories conditioned on a prospective treatment strategy.

#### Event-prediction heads

In contrast to state space models such as recurrent neural networks, attention-based models like SCOPE do not readily provide a hidden state vector that acts as a sufficient representation for the entire trajectory. Rather, the hidden vector produced at each time step is trained to predict the values of the clinical variables at the next time step. To circumvent this limitation, we condition our event predictions on several predicted hidden states.

We predict the score for a particular event, conditioned on history up to time *t* as

10 $${\hat{y}}_{t}={f}_{{{{\rm{event}}}}}([{z}_{t}^{(L)},{z}_{t+1}^{(L)},...,{z}_{t+K}^{(L)}]),$$

where *K* refers to the number of future hidden variables we are using in the event predictions. We found that using *K* = 1 leads to the best results in our experiments. Importantly, the prediction at time *t* does not use future values of the time series as ${z}_{t+k}^{(L)}$ only uses information up until *t*, as defined in Eq. (8). We used a different head for each type of event: disease progression, overall survival, and adverse events, corresponding to different functions ${f}_{{{{\rm{event}}}}}\in \{{f_{{{{\rm{PFS}}}}}}:{{\mathbb{R}}}^{{D}_{z}}\to {\mathbb{R}},{f_{{{{\rm{OS}}}}}}:{{\mathbb{R}}}^{{D}_{z}}\to {\mathbb{R}},{f_{{{{\rm{AE}}}}}}:{{\mathbb{R}}}^{{D}_{z}}\to {{\mathbb{R}}}^{12}\}$. These functions are all parametrized by neural networks.

#### Model training

The training of SCOPE consists of two steps - the first is pre-training the entire architecture on the forecasting task, whose objective function we specify below. The second step consisted of fine-tuning the model on only the event prediction task, which practically entailed training the final non-linear head that does the prediction of the event and freezing the remaining weights. We list the loss function for each step.

For the forecasting task, we trained the model auto-regressively, minimizing the discrepancy between the predicted values ${\widehat{X}}_{t+1}$ and true values *X*_*t*+1_. We used the observation mask *M* to only include the observed values in the loss function. Formally, the loss function for the forecasting objective is,

$$\begin{array}{r}{{{{\mathscr{L}}}}}_{{{{\rm{forecast}}}}}=\frac{1}{N}\mathop{\sum}\limits_{i}\frac{{\sum}_{t}{M}_{i}(t)\odot {({X}_{i}(t)-{\widehat{X}}_{i}(t))}^{2}}{{\sum}_{t}\sum {M}_{i}(t)}.\end{array}$$

For the fine-tuning step, we trained a linear head (or alternatively an MLP) for event prediction while freezing the remaining weights of the model. We used a Cox proportional hazards loss of the following form for training,

11 $${{{{\mathscr{L}}}}}_{{{{\rm{event}}}}}=\sum\limits_{t=1}^{{T}_{\max }}\frac{1}{{\sum }_{i}{{{\boldsymbol{1}}}}({y}_{i}\le t){{{\boldsymbol{1}}}}({{{\Delta }}}_{i}=1)}\sum\limits_{i:{{{\Delta }}}_{i}=1}{{{\boldsymbol{1}}}}({y}_{i}\le t)\left({\hat{y}}_{i}-\log \sum\limits_{j\in {{{\mathscr{R}}}}({y}_{i})}\exp ({\hat{y}}_{j})\right),$$

where Δ_*i*_ is the event indicator variable for patient *i*. Δ_*i*_ = 1 indicates that the event was observed for patient *i* and Δ_*i*_ = 0 indicates that the patient was censored. ${\hat{y}}_{i,t}$ is the predicted hazard rate outputted by the model for patient *i* at time *t*, and ${{{\mathcal{R}}}}$ is the set of patients still at risk for failure at time *t*. The event loss function can be defined for any event type: ${{{{\mathcal{L}}}}}_{{{{\rm{event}}}}}\in \{{{{{\mathcal{L}}}}}_{{{{\rm{PFS}}}}},{{{{\mathcal{L}}}}}_{{{{\rm{OS}}}}},{{{{\mathcal{L}}}}}_{{{{\rm{AE}}}}}\}$. We fine-tune the survival outcome (i.e., PFS or OS) and adverse event prediction heads independently with their corresponding loss functions. Both the forecasting and event prediction losses are minimized using the Adam optimizer^34^.

#### Bootstrap aggregation

To reduce the variance of our estimator and to provide reliable uncertainty predictions, we used bootstrap aggregation (also known as bagging), a gold standard machine learning technique for uncertainty estimation^35^. In practice, for each fold, we trained five different SCOPE models where the training set is composed of a random selection of the original training set (with replacement) sampled to 1.5 times the original size. The validation and test sets were left unchanged.

### Joint-modeling evaluation

We evaluated the performance of SCOPE on both event prediction and biomarker forecasting tasks. Evaluation was done on different durations of clinical history (*t*_cond_) and for different forecasting horizons (*t*_horizon_). We considered three observation windows and forecasting horizons (1, 6, and 12 months since most patients experienced the event or were censored after two years). Our method supports an arbitrary duration for the available clinical history (*t*_cond_) and can thus use the entirety of the available clinical history of a patient, regardless of its specific duration. For each individual prediction task, we compared SCOPE against methods trained specifically to that particular task and to a particular duration of the clinical history. Intuitively, this experimental setup advantages the task-specialized methods, and thus represents a strong benchmark for the evaluation of our model.

#### Event prediction baselines

We compared against four baseline models. Two standard survival models, a Cox Proportional Hazards (CPH) model and a Random Survival Forest (RSF), were used as baseline approaches for survival prediction (PFS, OS) as well as adverse event prediction. These are standard survival models that have been widely used in a wide variety of risk prediction tasks across several chronic diseases^4,17–19^. Note that a separate baseline model was trained for each observation window length, *t*_cond_ (e.g., separate CPH models were trained for each *t*_cond_). To form the input vector of these methods, for an observation window of *t* time steps, we concatenated the observations at the last time step (*X*(*t*)), the observations at the first time step (*X*(0)), and the baseline variables (*B*). Feeding both *X*(*t*) and *X*(0) to the model provides information about the trajectory of the patient.

Our third baseline is a CPH model where the only covariate that is inputted to the model is the ISS stage of a patient, a common risk score used in multiple myeloma that is computed from a patient’s serum beta-2 microglobulin and serum albumin values and ranges from one to three^36^. This model is denoted as CPH-ISS. CPH-ISS can be seen as the “standard of care” approach used by oncologists to stratify myeloma patients according to their risk. Naturally, this baseline is only relevant for survival prediction (and not adverse event prediction), since the particular biomarkers used to compute the risk score are largely independent of the adverse events.

Our last baseline is a Dynamic-DeepHit model that takes the same inputs as SCOPE but relies on a recurrent neural network architecture and frames the time-to-event prediction as a multiclass classification objective^16^. This model is denoted as DDH. We used the Pytorch implementation available at https://github.com/Jeanselme/DynamicDeepHit. In our experiments, we searched across different configurations of the hidden dimension, i.e., [16, 32, 64], for DDH, which is comparable to the hidden dimension search used for SCOPE.

#### Forecasting baselines

We compare the performance of our method against two state-of-the-art time series forecasting methods (recurrent neural networks and deep Markov models) and a standard imputation method (last observation carried forward). Except for the last observation carried forward method, the models use the same inputs and are trained on the same reconstruction task as SCOPE. However, these models do not predict the risk of future events.

#### Statistical analysis

*p* values for specific pairwise comparisons between methods (for a given *t*_cond_, *t*_horizon_, and dataset) were obtained using pairwise independent t-tests with 4 d.f. Overall comparisons between methods were performed using a two-way analysis of variance (ANOVA) without an interaction term, where the first factor was set as the method and the second factor was the combination of *t*_cond_, *t*_horizon_, and dataset. We correct for multiple testing across the different pairs of methods using a Bonferroni correction. The resulting *p* values for specific and general pairwise comparisons are available in the Supplementary Material (Supplementary Tables 12–18).

##### Recurrent neural networks

RNNs are a widely used class of neural networks for time series processing. They operate by maintaining an internal memory state that is updated as the network processes each input in the sequence. The memory state is passed from one step of the sequence to the next, allowing the network to “remember” previous inputs and use them to inform the processing of future inputs. At each step of the sequence, the RNN takes in an input and combines it with the current memory state to generate a new memory state and an output. Unlike our approach, which relies on attention-based models, RNN process the time series *sequentially*. This restricts their ability to efficiently capture long-term dependencies.

##### Deep Markov Models

Deep Markov Models (DMMs) are a class of probabilistic models dedicated to time series modeling. DMMs take their name from the Markov assumption, which states that the current state of a system depends only on its previous state, and not on the entire history. DMMs extend this idea to deep architectures by adding multiple hidden layers, allowing them to capture complex dependencies between inputs. Unlike RNNs, which process sequences in a deterministic manner, DMMs are stochastic models that can generate multiple possible outputs for a given input. This makes them well-suited for modeling noisy time series data. Akin to RNNs, DMMs process the temporal observation sequentially, which limits their ability to capture long-term dependencies.

##### Last Observation Carried Forward

Last Observation Carried Forward (LOCF) is a simple imputation method used in time series forecasting. LOCF replaces missing values in a time series with the most recent available observation. LOCF is a straightforward and computationally inexpensive approach, making it a popular choice in many applications. However, LOCF does not consider the temporal dependencies between observations and may not capture the underlying patterns and dynamics in the time series data. In contrast, RNNs, DMMs, or attention-based models like SCOPE are designed to work with sequential data and can capture complex temporal dependencies.

### SCOPE introspection

We performed introspection of the transformer hidden states in SCOPE. The hidden states are “joint” representations that are used to predict adverse events, survival, and biomarkers longitudinally. The hidden representations at each time *t* are denoted as ${z}_{t}^{(L)}$. The predictions for disease progression and biomarker forecasting are referred to as ${\hat{y}}_{t,{{{\rm{pfs}}}}}\in {\mathbb{R}}$ and ${\widehat{X}}_{t}$, respectively. We obtain the UMAP plot in Fig. 3a by using the the ${z}_{1}^{(L)}$ vectors (at the first time step) of each patient.

The experiment reported in Fig. 3b consists of computing the Pearson correlation (*ρ*) between the different dimensions of the hidden states ${z}_{t}^{(L)}$ and the various predictions:

- For PFS, we have $\rho ({z}_{t,d}^{(L)},{\hat{y}}_{t,{{{\rm{pfs}}}}}),\forall d\in \{1,\ldots ,{D}_{z}\}$.
- For each biomarker variable *j*, we have $\rho ({z}_{t,d},{\widehat{X}}_{t,j}),\forall d\in \{1,\ldots ,{D}_{z}\}$.

These correlations were computed for *t* equal to 1, 3, 6, 9, and 12 months.

We also computed a more interpretable risk score for each patient that represents the risk of their experiencing an event. Although the output of the event-prediction head represents such a score in theory, these scores are not limited in range and are only informative relative to the scores of other patients. Indeed, the semi-parametric nature of proportional hazards models only enforces a consistent ordering of the scores across the dataset. The absolute value of the score is not meaningful. Thus, to improve the interpretability of the event prediction scores, we compute a normalized score $\tilde{y}$:

12 $$\begin{array}{r}{\tilde{y}}_{t}=\frac{{\hat{y}}_{t}-{\hat{y}}_{min}}{{\hat{y}}_{min}-{\hat{y}}_{max}},\end{array}$$

where ${\hat{y}}_{min}$ and ${\hat{y}}_{max}$ are the minimum and maximum values of the score across patients and time in the training set. This normalized score can directly be interpreted as a quantile of the risk of a particular patient with respect to the whole cohort. For instance, ${\tilde{y}}_{t}=0.7$ suggests that 70% of patients in the cohort have a lower risk score. We used these normalized scores in Fig. 3.

### Treatment effect estimation and discovering heterogeneous subgroups

We used a trained SCOPE model to impute the counterfactuals on the entire MM2 dataset. Counterfactuals were obtained by using our model to perform predictions, with modified treatment input vectors. Information about the treatment appears both in vector *B* and in the longitudinal treatment vector *A*. We thus changed both the treatment arm covariate in *B* as well as the dosages in *A* to the desired treatment regimen, i.e., either Rd or IRd, before doing inference. We performed predictions of potential outcomes at baseline only (as this is the only time step where randomization occurs). We write the potential outcomes, i.e., the predicted PFS risk score, under IRd and Rd, respectively:

$$\begin{array}{r}{\hat{y}}_{{{{\rm{PFS}}}}}^{1}={f_{PFS}}({z}_{0}^{(L)}({X}_{0},{B}^{1},{A}^{1}))\\ {\hat{y}}_{{{{\rm{PFS}}}}}^{0}={f_{PFS}}({z}_{0}^{(L)}({X}_{0},{B}^{0},{A}^{0})).\end{array}\,$$

*B*^1^ and *B*^0^ refer to the baseline vectors where the treatment indicator is set to 1 or 0. Similarly, *A*^1^ and *A*^0^ refer to the IRd and Rd treatment strategies, respectively. Finally, we define potential biomarker trajectories under the two treatment regimens:

$$\begin{array}{r}{\widehat{X}}_{t,{{{\rm{PFS}}}}}^{1}={f_{{{{\rm{pred}}}}}}({z}_{0+t}^{(L)}({X}_{0},{B}^{1},{A}^{1}))\\ {\widehat{X}}_{t,{{{\rm{PFS}}}}}^{0}={f_{{{{\rm{pred}}}}}}({z}_{0+t}^{(L)}({X}_{0},{B}^{0},{A}^{0})),\end{array}$$

where we make explicit that the trajectories are only conditioned on information available at baseline. Examples of biomarkers trajectories conditioned on different treatments are presented in Fig. 4a.

Given ${\hat{y}}_{{{{\rm{PFS}}}}}^{1}$ and ${\hat{y}}_{{{{\rm{PFS}}}}}^{0}$, we can define a conditional average treatment effect (CATE) on risk of disease progression for each patient as follows,

$$\begin{array}{r}{{{\rm{CATE}}}}={\hat{y}}_{{{{\rm{PFS}}}}}^{1}-{\hat{y}}_{{{{\rm{PFS}}}}}^{1}.\end{array}$$

*p* values in subgroup analysis are highly impacted by the sample size. To provide more reliable estimates, we created an even split of the MM2 cohort into a train and test set. This new train set consisted fully of the original train set minus a random selection patients to reach 50% of the cohort. The rest of the patients then comprised the test set. All model training and policy learning are done on the train set, and all Kaplan-Meier curves shown are computed on the test set.

Using this training cohort from MM2, we then learned a policy to assign treatment; specifically, we used the median conditional average treatment effect (CATE) over the training patients as a threshold to determine whether a patient would be assigned to a subgroup for whom IRd would be most efficacious. Because $\hat{y}$ represents the *risk* of disease progression, a positive CATE indicates a negative effect of IRd (the risk of disease progression is higher) while a negative CATE indicates a positive effect of IRd. We then defined the subgroup threshold as the median of all CATE values in the training set ($\delta ={{{{\rm{CATE}}}}}_{\frac{1}{2}}$). We obtained the initial subgroup assignment strategy, π*, to determine if someone is included in the subgroup or not, as follows, for each patient *i*:

$$\begin{array}{r}{\pi }_{i}^{* }=\left\{\begin{array}{l}0\quad {{{\rm{if}}}}\quad {{{{\rm{CATE}}}}}_{i} \,> \,\delta \quad \\ 1\quad {{{\rm{if}}}}\quad {{{{\rm{CATE}}}}}_{i}\,\le \,\delta \quad \\ \end{array}\right.\end{array}$$

Intuitively, a patient is in the subgroup, i.e., π* = 1, if IRd is effective at decreasing their risk of progression.

To improve the interpretability of this subgroup assignment strategy, we learned a shallow decision tree where we used ${\pi }_{i}^{* },\forall i$ as the labels. We trained the decision tree on the training cohort using the baseline clinical variables, *B*, as covariates. The output of this decision tree was then used as another, more interpretable, subgroup strategy, *π*. Kaplan–Meier curves were estimated for the discovered subgroups (found via both subgroup assignment strategies, π^*^ and *π*) by looking at the actual treatment and control groups in the subgroup (Fig. 4c).

### Supplementary information

Supplemental Material

## Data Availability

The data used in this study are derived from proprietary clinical trials run by Takeda Pharmaceuticals and thus are not made publicly available due to data privacy concerns. Reasonable requests for collaboration using the data can be made from the authors, as feasible and permitted by Takeda Pharmaceuticals. Takeda has made the anonymized patient-level data available to bona fide investigators through the data sharing portal http://vivli.org, studies ID NCT01850524 and NCT01564537.

## Code availability

All model code and algorithms are available at the following GitHub repository: https://github.com/clinicalml/SCOPE.
