# Supplementary material for: Joint AI-driven event prediction and longitudinal modeling in newly diagnosed and relapsed multiple myeloma
Source: NPJ Digit Med. 2024 Jul 29;7:200. doi: 10.1038/s41746-024-01189-3 (PMC11286964; doi:10.1038/s41746-024-01189-3)
Supplement: Supplementary file 1 — Supplemental Material [file 41746_2024_1189_MOESM1_ESM.pdf]

Joint AI-driven event prediction and longitudinal modeling in newly diagnosed and relapsed multiple myeloma — Supplementary Material

Zeshan Hussain[*,1,2], Edward De Brouwer[*,1], Rebecca Boiarsky[1], Sama Setty[1], Neeraj Gupta[3], Guohui Liu[3], Cong Li[3], Jaydeep Srimani[3], Jacob Zhang[3], Rich Labotka[3], and David Sontag[,1]**

[1]CSAIL, MIT, Cambridge, USA

[2]Harvard Medical School, Boston, USA

[3]Takeda, LLC

[*]these authors contributed equally to this work

[**]corresponding author: dsontag@csail.mit.edu

ABSTRACT

Multiple myeloma management requires a balance between maximizing survival, minimizing adverse events to therapy, and monitoring disease progression. While previous work has proposed data-driven models for individual tasks, these approaches fail to provide a holistic view of a patient's disease state, limiting their utility to assist physician decision-making. To address this limitation, we developed a transformer-based machine learning model that jointly (1) predicts progression-free survival (PFS), overall survival (OS), and adverse events (AE), (2) forecasts key disease biomarkers, and (3) assesses the effect of different treatment strategies, e.g. ixazomib, lenalidomide, dexamethasone (IRd) vs lenalidomide, dexamethasone (Rd). Using TOURMALINE trial data, we trained and internally validated our model on newly diagnosed myeloma patients ($N = 703$) and externally validated it on relapsed and refractory myeloma patients ($N = 720$). Our model achieved superior performance to a risk model based on the multiple myeloma international staging system (ISS) ($p < 0.001$, *Bonferroni corrected*) and comparable performance to survival models trained separately on each task, but unable to forecast biomarkers. Our approach outperformed state-of-the-art deep learning models, tailored towards forecasting, on predicting key disease biomarkers ($p < 0.001$, *Bonferroni corrected*). Finally, leveraging our model's capacity to estimate individual-level treatment effects, we found that patients with IgA kappa myeloma appear to benefit the most from IRd. Our study suggests that a holistic assessment of a patient's myeloma course is possible, potentially serving as the foundation for a personalized decision support system.

Supplementary Material

Additional Quantitative Results

In Supplementary Figure 1, we further introspect into the learned hidden states of our model by plotting the correlations between the hidden states and acute renal failure as well as GFR. We saw that hidden dimensions indicating higher risk of acute renal failure also indicated lower GFR levels, aligning with clinical intuition.

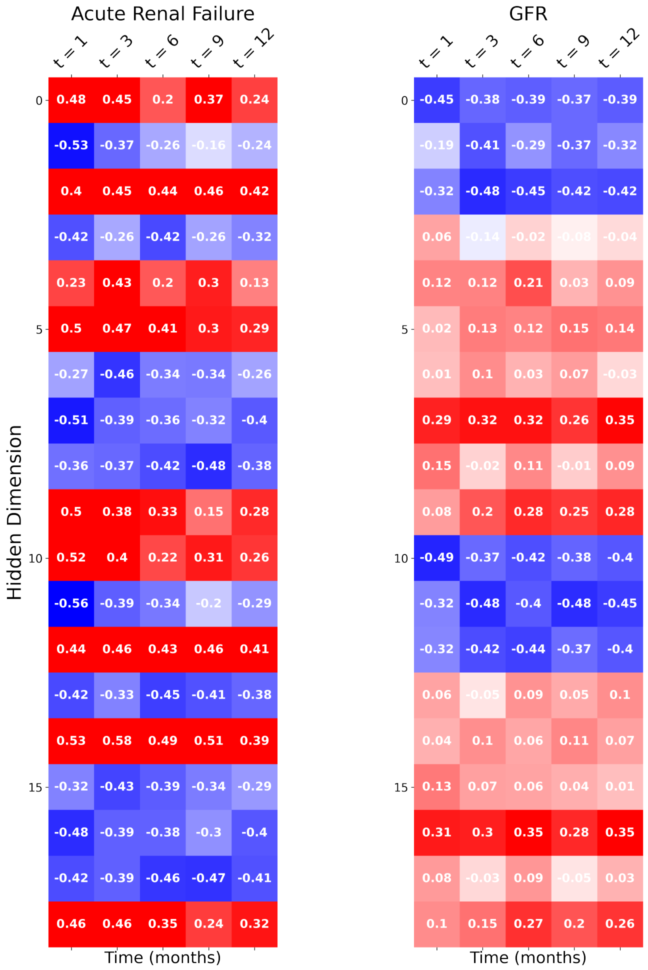

Supplementary Figure 1. Additional model introspection. At five different time points, we computed the correlation between the hidden state for all patients and the risk of acute renal failure (*predicted adverse event*), and GFR (*feature*), respectively. The number of hidden dimensions is 64, but only the dimensions that had at least one time point above 0.4 were shown. Red indicates a positive association between the hidden state value and the feature or prediction, whereas blue indicates a negative association. We observed that in dimensions where there was a growing risk of acute renal failure, GFR levels tended to go down, aligning with clinical intuition.

In Supplementary Figure 2, we graphically depict the performance of the different methods on PFS and OS predictions. We also report the quantitative results of our experiments on biomarker forecasting (Supplementary Table 11), PFS prediction (Supplementary Tables 1, 2, and 3), OS prediction (Supplementary Tables 4, 5, and 6), and AE prediction (Supplementary Tables 7, 8, 9, and 10). We report the results of pairwise two-sample t-tests with 4 d.f. for difference of means between different methods in Supplementary Tables 12, 13, 14, 15, 16, and 17. We

report the overall methods comparisons with two-way ANOVA in Supplementary Tables 18 and 19. In Supplementary Figure 3, we plot the difference between the next observation and the current observation (accounting for missing values) aggregated over the different patients and for different sets of variables (all, serum immunoglobulins, and chemistry labs). We observed that the longitudinal variables tend to stabilize around a fixed value over time, making the forecasting task easier over longer horizons.

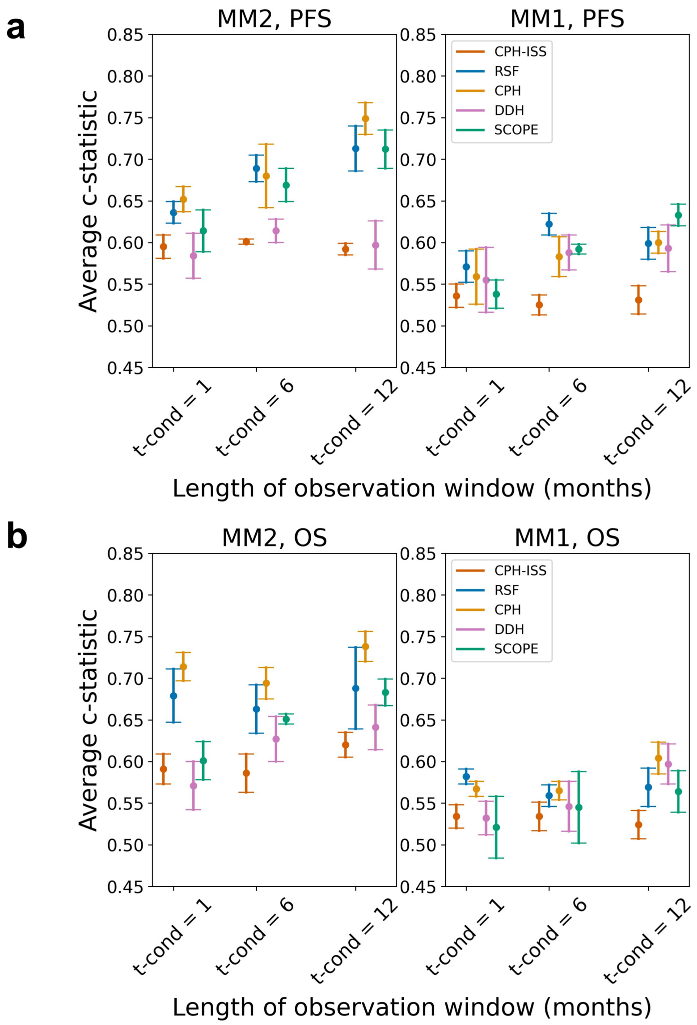

Supplementary Figure 2. PFS and OS prediction performance on MM1 and MM2 cohorts. **(A)**: We report concordance index based on inverse probability of censoring weights (C-index IPCW) averaged across three time quantiles (25th, 50th, and 75th quantiles) at different observation windows (1 month, 6 months, and 12 months), for the MM1 and MM2 cohorts, on the progression free survival prediction task. **(B)**: We report concordance index based on inverse probability of censoring weights (C-index IPCW) averaged across three time quantiles (25th, 50th, and 75th quantiles) at different observation windows (1 month, 6 months, and 12 months), for the MM1 and MM2 cohorts, on the overall survival prediction task.

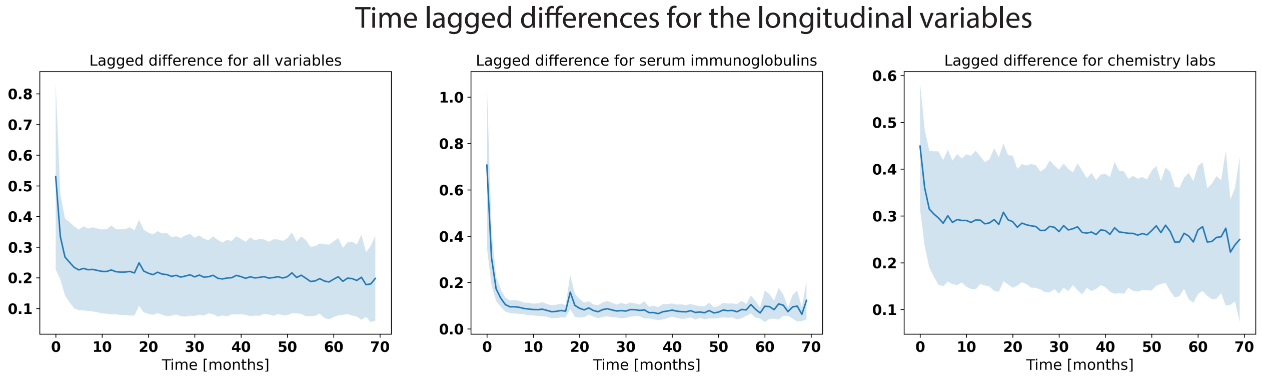

Supplementary Figure 3. Time-lagged differences in the longitudinal variables. At each time step, we compute the difference between the subsequent observation and the current one (accounting for missing values). We observe that the longitudinal variables tend to stabilize over time, making forecasting easier over longer horizons.

	MM2			MM1		
	1	6	12	1	6	12
RSF	0.636 ± 0.013	0.689 ± 0.016	0.713 ± 0.027	0.571 ± 0.019	0.622 ± 0.013	0.599 ± 0.019
CPH	0.652 ± 0.015	0.680 ± 0.038	0.749 ± 0.019	0.559 ± 0.033	0.583 ± 0.024	0.600 ± 0.013
CPH-ISS	0.595 ± 0.014	0.601 ± 0.003	0.592 ± 0.007	0.536 ± 0.014	0.525 ± 0.012	0.531 ± 0.017
DDH	0.584 ± 0.027	0.614 ± 0.014	0.597 ± 0.029	0.555 ± 0.039	0.588 ± 0.021	0.593 ± 0.028
SCOPE	0.614 ± 0.025	0.669 ± 0.002	0.712 ± 0.023	0.538 ± 0.017	0.592 ± 0.006	0.633 ± 0.013

Supplementary Table 1. PFS prediction results for all patients, t_{cond} in the columns

	MM2			MM1		
	1	6	12	1	6	12
RSF	0.673 ± 0.050	0.685 ± 0.058	0.676 ± 0.037	0.536 ± 0.014	0.576 ± 0.024	0.627 ± 0.031
CPH	0.632 ± 0.031	0.710 ± 0.041	0.639 ± 0.045	0.569 ± 0.022	0.572 ± 0.022	0.632 ± 0.023
CPH-ISS	0.583 ± 0.027	0.600 ± 0.006	0.592 ± 0.009	0.550 ± 0.024	0.498 ± 0.021	0.561 ± 0.016
DDH	0.547 ± 0.110	0.625 ± 0.035	0.486 ± 0.067	0.538 ± 0.045	0.546 ± 0.025	0.643 ± 0.037
SCOPE	0.616 ± 0.029	0.589 ± 0.025	0.654 ± 0.039	0.550 ± 0.026	0.565 ± 0.012	0.678 ± 0.040

Supplementary Table 2. PFS prediction results for IgA-dominant myeloma subgroup, t_{cond} in the columns

	MM2			MM1		
	1	6	12	1	6	12
RSF	0.637 ± 0.021	0.699 ± 0.019	0.76 ± 0.033	0.592 ± 0.015	0.629 ± 0.017	0.614 ± 0.018
CPH	0.654 ± 0.017	0.693 ± 0.040	0.801 ± 0.025	0.564 ± 0.056	0.587 ± 0.03	0.608 ± 0.014
CPH-ISS	0.592 ± 0.009	0.596 ± 0.001	0.593 ± 0.007	0.550 ± 0.019	0.550 ± 0.009	0.536 ± 0.015
DDH	0.572 ± 0.056	0.619 ± 0.026	0.624 ± 0.049	0.537 ± 0.052	0.614 ± 0.029	0.594 ± 0.025
SCOPE	0.639 ± 0.018	0.709 ± 0.025	0.746 ± 0.028	0.618 ± 0.006	0.621 ± 0.005	0.623 ± 0.018

Supplementary Table 3. PFS prediction results for IgG-dominant myeloma subgroup, t_{cond} in the columns

	MM2			MM1		
t_{cond}	1	6	12	1	6	12
RSF	0.679 ± 0.032	0.663 ± 0.029	0.688 ± 0.049	0.582 ± 0.009	0.559 ± 0.013	0.569 ± 0.023
CPH	0.714 ± 0.017	0.694 ± 0.019	0.738 ± 0.018	0.567 ± 0.009	0.565 ± 0.011	0.604 ± 0.019
CPH-ISS	0.591 ± 0.018	0.586 ± 0.023	0.620 ± 0.015	0.534 ± 0.014	0.534 ± 0.017	0.524 ± 0.017
DDH	0.571 ± 0.029	0.627 ± 0.027	0.641 ± 0.027	0.532 ± 0.020	0.546 ± 0.030	0.597 ± 0.024
SCOPE	0.601 ± 0.023	0.651 ± 0.006	0.683 ± 0.016	0.521 ± 0.037	0.545 ± 0.043	0.564 ± 0.025

Supplementary Table 4. OS prediction results for all patients, t_{cond} in the columns

	MM2			MM1		
t_{cond}	1	6	12	1	6	12
RSF	0.656 ± 0.062	0.574 ± 0.056	0.664 ± 0.084	0.549 ± 0.024	0.504 ± 0.015	0.577 ± 0.023
CPH	0.674 ± 0.040	0.588 ± 0.06	0.573 ± 0.104	0.561 ± 0.011	0.54 ± 0.015	0.678 ± 0.026
CPH-ISS	0.533 ± 0.051	0.533 ± 0.051	0.586 ± 0.064	0.547 ± 0.023	0.514 ± 0.030	0.555 ± 0.016
DDH	0.580 ± 0.061	0.622 ± 0.076	0.601 ± 0.036	0.514 ± 0.033	0.529 ± 0.031	0.630 ± 0.052
SCOPE	0.630 ± 0.028	0.562 ± 0.029	0.615 ± 0.027	0.503 ± 0.042	0.499 ± 0.064	0.604 ± 0.054

Supplementary Table 5. OS prediction results for IgA-dominant myeloma subgroup, t_{cond} in the columns

	MM2			MM1		
t_{cond}	1	6	12	1	6	12
RSF	0.641 ± 0.040	0.688 ± 0.029	0.701 ± 0.057	0.579 ± 0.015	0.557 ± 0.015	0.557 ± 0.034
CPH	0.670 ± 0.024	0.681 ± 0.029	0.749 ± 0.014	0.593 ± 0.008	0.570 ± 0.010	0.571 ± 0.021
CPH-ISS	0.602 ± 0.027	0.589 ± 0.029	0.601 ± 0.018	0.547 ± 0.019	0.558 ± 0.013	0.530 ± 0.015
DDH	0.584 ± 0.037	0.629 ± 0.026	0.655 ± 0.033	0.511 ± 0.048	0.530 ± 0.055	0.564 ± 0.024
SCOPE	0.580 ± 0.021	0.670 ± 0.005	0.685 ± 0.011	0.540 ± 0.062	0.552 ± 0.050	0.546 ± 0.015

Supplementary Table 6. OS prediction results for IgG-dominant myeloma subgroup, t_{cond} in the columns

	AE-0	AE-1	AE-2	AE-3	AE-4	AE-5
RSF	0.590 ± 0.080	0.616 ± 0.048	0.499 ± 0.062	0.484 ± 0.118	0.568 ± 0.092	0.386 ± 0.262
CPH	0.626 ± 0.021	0.505 ± 0.106	0.468 ± 0.036	0.452 ± 0.069	0.631 ± 0.063	0.471 ± 0.149
DDH	0.515 ± 0.059	0.521 ± 0.087	0.462 ± 0.041	0.604 ± 0.050	0.540 ± 0.166	0.497 ± 0.217
SCOPE	0.607 ± 0.030	0.533 ± 0.132	0.501 ± 0.054	0.371 ± 0.134	0.550 ± 0.121	0.463 ± 0.223

Supplementary Table 7. Prediction of Adverse Events at $t_{\text{cond}} = 1$ month. AE 0-5.

	AE-6	AE-7	AE-8	AE-9	AE-10	AE-11
RSF	0.534 ± 0.078	0.531 ± 0.055	0.729 ± 0.099	0.474 ± 0.047	0.589 ± 0.111	0.454 ± 0.096
CPH	0.616 ± 0.112	0.564 ± 0.022	0.756 ± 0.09	0.495 ± 0.046	0.694 ± 0.092	0.504 ± 0.066
DDH	0.461 ± 0.130	0.569 ± 0.081	0.636 ± 0.153	0.547 ± 0.055	0.589 ± 0.100	0.542 ± 0.075
SCOPE	0.658 ± 0.098	0.473 ± 0.097	0.594 ± 0.102	0.521 ± 0.073	0.623 ± 0.114	0.459 ± 0.132

Supplementary Table 8. Prediction of Adverse Events at $t_{\text{cond}} = 1$ month. AE 6-11.

	AE-0	AE-1	AE-2	AE-3	AE-4	AE-5
RSF	0.584 ± 0.092	0.586 ± 0.124	0.436 ± 0.047	0.661 ± 0.157	0.483 ± 0.134	0.429 ± 0.169
CPH	0.663 ± 0.042	0.520 ± 0.106	0.472 ± 0.029	0.629 ± 0.111	0.360 ± 0.212	0.310 ± 0.190
DDH	0.489 ± 0.100	0.534 ± 0.111	0.483 ± 0.052	0.551 ± 0.091	0.336 ± 0.346	0.628 ± 0.279
SCOPE	0.653 ± 0.092	0.544 ± 0.190	0.468 ± 0.056	0.580 ± 0.106	0.596 ± 0.191	0.369 ± 0.335

Supplementary Table 9. Prediction of Adverse Events at $t_{\text{cond}} = 6$ months. AE 0-5.

	AE-6	AE-7	AE-8	AE-9	AE-10	AE-11
RSF	0.471 ± 0.247	0.644 ± 0.036	0.437 ± 0.230	0.454 ± 0.107	0.676 ± 0.109	0.563 ± 0.127
CPH	0.441 ± 0.161	0.636 ± 0.054	0.229 ± 0.197	0.415 ± 0.048	0.929 ± 0.037	0.621 ± 0.121
DDH	0.485 ± 0.177	0.671 ± 0.120	0.671 ± 0.154	0.424 ± 0.186	0.728 ± 0.168	0.431 ± 0.115
SCOPE	0.523 ± 0.165	0.570 ± 0.067	0.560 ± 0.367	0.483 ± 0.047	0.861 ± 0.130	0.431 ± 0.208

Supplementary Table 10. Prediction of Adverse Events at $t_{\text{cond}} = 6$ months. AE 6-11.

t_{cond}		1	6	12
all	LOCF	1.189 ± 0.000	0.245 ± 0.000	0.220 ± 0.000
	DMM	0.804 ± 0.038	0.614 ± 0.013	0.533 ± 0.019
	RNN	0.429 ± 0.038	0.263 ± 0.007	0.231 ± 0.010
	SCOPE	0.341 ± 0.011	0.208 ± 0.002	0.181 ± 0.003
chem	LOCF	0.392 ± 0.000	0.287 ± 0.000	0.260 ± 0.000
	DMM	0.676 ± 0.018	0.649 ± 0.015	0.588 ± 0.006
	RNN	0.338 ± 0.010	0.285 ± 0.004	0.254 ± 0.010
	SCOPE	0.278 ± 0.005	0.241 ± 0.003	0.207 ± 0.003
serum	LOCF	2.975 ± 0.000	0.209 ± 0.000	0.124 ± 0.000
	DMM	1.784 ± 0.192	0.949 ± 0.113	0.750 ± 0.067
	RNN	0.879 ± 0.157	0.312 ± 0.027	0.267 ± 0.044
	SCOPE	0.660 ± 0.038	0.193 ± 0.004	0.166 ± 0.008

Supplementary Table 11. Forecasting performance (MSE) for different methods with horizon 12 months.

t_{cond}		1				6				12			
Method		LOCF	DMM	RNN	SCOPE	LOCF	DMM	RNN	SCOPE	LOCF	DMM	RNN	SCOPE
all	LOCF	1.0	<1e-4	<1e-4	<1e-4	1.0	<1e-4	0.0045	<1e-4	1.0	<1e-4	0.0697	<1e-4
	DMM	<1e-4	1.0	0.0001	<1e-4	<1e-4	1.0	<1e-4	<1e-4	<1e-4	1.0	<1e-4	<1e-4
	RNN	<1e-4	0.0001	1.0	0.0076	0.0045	<1e-4	1.0	0.0001	0.0697	<1e-4	1.0	0.0004
	SCOPE	<1e-4	<1e-4	0.0076	1.0	<1e-4	<1e-4	0.0001	1.0	<1e-4	<1e-4	0.0004	1.0
chem	LOCF	1.0	<1e-4	0.0003	<1e-4	1.0	<1e-4	0.3262	<1e-4	1.0	<1e-4	0.2508	<1e-4
	DMM	<1e-4	1.0	<1e-4	<1e-4	<1e-4	1.0	<1e-4	<1e-4	<1e-4	1.0	<1e-4	<1e-4
	RNN	0.0003	<1e-4	1.0	0.0003	0.3262	<1e-4	1.0	<1e-4	0.2508	<1e-4	1.0	0.0005
	SCOPE	<1e-4	<1e-4	0.0003	1.0	<1e-4	<1e-4	<1e-4	1.0	<1e-4	<1e-4	0.0005	1.0
serum	LOCF	1.0	0.0002	<1e-4	<1e-4	1.0	0.0001	0.001	0.0009	1.0	<1e-4	0.0019	0.0003
	DMM	0.0002	1.0	0.0012	0.0002	0.0001	1.0	0.0003	0.0001	<1e-4	1.0	0.0002	<1e-4
	RNN	<1e-4	0.0012	1.0	0.0387	0.001	0.0003	1.0	0.0006	0.0019	0.0002	1.0	0.0072
	SCOPE	<1e-4	0.0002	0.0387	1.0	0.0009	0.0001	0.0006	1.0	0.0003	<1e-4	0.0072	1.0

Supplementary Table 12. p-values from pairwise t-tests with 4 $d.f.$ for difference of means between different methods in the biomarker forecasting with $t_{\text{horizon}} = 12$. We stratified the tests per groups of variables and per t_{cond}.

t_{cond}		1				6				12			
Method		LOCF	DMM	RNN	SCOPE	LOCF	DMM	RNN	SCOPE	LOCF	DMM	RNN	SCOPE
all	LOCF	1.0	0.5516	<1e-4	<1e-4	1.0	<1e-4	0.0019	<1e-4	1.0	<1e-4	0.606	<1e-4
	DMM	0.5516	1.0	<1e-4	<1e-4	<1e-4	1.0	<1e-4	<1e-4	<1e-4	1.0	<1e-4	<1e-4
	RNN	<1e-4	<1e-4	1.0	0.0043	0.0019	<1e-4	1.0	<1e-4	0.606	<1e-4	1.0	0.0001
	SCOPE	<1e-4	<1e-4	0.0043	1.0	<1e-4	<1e-4	<1e-4	1.0	<1e-4	<1e-4	0.0001	1.0
chem	LOCF	1.0	<1e-4	0.0022	<1e-4	1.0	<1e-4	0.0002	<1e-4	1.0	<1e-4	0.0032	<1e-4
	DMM	<1e-4	1.0	<1e-4	<1e-4	<1e-4	1.0	<1e-4	<1e-4	<1e-4	1.0	<1e-4	<1e-4
	RNN	0.0022	<1e-4	1.0	0.0001	0.0002	<1e-4	1.0	0.0001	0.0032	<1e-4	1.0	0.0007
	SCOPE	<1e-4	<1e-4	0.0001	1.0	<1e-4	<1e-4	0.0001	1.0	<1e-4	<1e-4	0.0007	1.0
serum	LOCF	1.0	0.5092	<1e-4	<1e-4	1.0	<1e-4	0.0011	0.4216	1.0	<1e-4	0.0009	0.0001
	DMM	0.5092	1.0	<1e-4	<1e-4	<1e-4	1.0	<1e-4	<1e-4	<1e-4	1.0	<1e-4	<1e-4
	RNN	<1e-4	<1e-4	1.0	0.0849	0.0011	<1e-4	1.0	0.0011	0.0009	<1e-4	1.0	0.0033
	SCOPE	<1e-4	<1e-4	0.0849	1.0	0.4216	<1e-4	0.0011	1.0	0.0001	<1e-4	0.0033	1.0

Supplementary Table 13. p-values from pairwise t-tests with 4 $d.f.$ for difference of means between different methods in the biomarker forecasting with $t_{\text{horizon}} = 6$. We stratified the tests per groups of variables and per t_{cond}.

t_{cond}		1					6					12				
		RSF	CPH	CPH-ISS	DDH	SCOPE	RSF	CPH	CPH-ISS	DDH	SCOPE	RSF	CPH	CPH-ISS	DDH	SCOPE
MM2	RSF	1.0000	0.1458	0.0087	0.0178	0.1558	1.0000	0.6510	0.0003	0.0014	0.1557	1.0000	0.0713	0.0006	0.0028	0.9528
	CPH	0.1458	1.0000	0.0034	0.0079	0.0435	0.6510	1.0000	0.0098	0.0219	0.5974	0.0713	1.0000	0.0001	0.0006	0.0502
	CPH-ISS	0.0087	0.0034	1.0000	0.4640	0.2123	0.0003	0.0098	1.0000	0.1122	0.0017	0.0006	0.0001	1.0000	0.7269	0.0004
	DDH	0.0178	0.0079	0.4640	1.0000	0.1424	0.0014	0.0219	0.1122	1.0000	0.0073	0.0028	0.0006	0.7269	1.0000	0.0023
	SCOPE	0.1558	0.0435	0.2123	0.1424	1.0000	0.1557	0.5974	0.0017	0.0073	1.0000	0.9528	0.0502	0.0004	0.0023	1.0000
MM1	RSF	1.0000	0.5199	0.0295	0.4559	0.0444	1.0000	0.0331	0.0003	0.0370	0.0094	1.0000	0.9273	0.0040	0.7120	0.0299
	CPH	0.5199	1.0000	0.2247	0.8695	0.2746	0.0331	1.0000	0.0084	0.7436	0.4616	0.9273	1.0000	0.0020	0.6388	0.0159
	CPH-ISS	0.0295	0.2247	1.0000	0.3632	0.8490	0.0003	0.0084	1.0000	0.0043	0.0004	0.0040	0.0020	1.0000	0.0133	0.0004
	DDH	0.4559	0.8695	0.3632	1.0000	0.4221	0.0370	0.7436	0.0043	1.0000	0.7031	0.7120	0.6388	0.0133	1.0000	0.0442
	SCOPE	0.0444	0.2746	0.8490	0.4221	1.0000	0.0094	0.4616	0.0004	0.7031	1.0000	0.0299	0.0159	0.0004	0.0442	1.0000

Supplementary Table 14. p-values from pairwise t-tests with 4 $d.f.$ for difference of means between different methods in the PFS prediction. We stratified the tests per study and per t_{cond}.

t_{cond}		1					6					12				
		RSF	CPH	CPH-ISS	DDH	SCOPE	RSF	CPH	CPH-ISS	DDH	SCOPE	RSF	CPH	CPH-ISS	DDH	SCOPE
MM2	RSF	1.0000	0.0969	0.0058	0.0050	0.0115	1.0000	0.1162	0.0096	0.1120	0.4161	1.0000	0.0989	0.0413	0.1335	0.8389
	CPH	0.0969	1.0000	0.0004	0.0007	0.0009	0.1162	1.0000	0.0013	0.0105	0.0085	0.0989	1.0000	0.0004	0.0026	0.0070
	CPH-ISS	0.0058	0.0004	1.0000	0.2603	0.4866	0.0096	0.0013	1.0000	0.0610	0.0036	0.0413	0.0004	1.0000	0.2031	0.0030
	DDH	0.0050	0.0007	0.2603	1.0000	0.1442	0.1120	0.0105	0.0610	1.0000	0.1243	0.1335	0.0026	0.2031	1.0000	0.0402
	SCOPE	0.0115	0.0009	0.4866	0.1442	1.0000	0.4161	0.0085	0.0036	0.1243	1.0000	0.8389	0.0070	0.0030	0.0402	1.0000
MM1	RSF	1.0000	0.0579	0.0030	0.0070	0.0231	1.0000	0.4749	0.0593	0.4242	0.5243	1.0000	0.0586	0.0245	0.1327	0.7586
	CPH	0.0579	1.0000	0.0114	0.0234	0.0540	0.4749	1.0000	0.0267	0.2544	0.3707	0.0586	1.0000	0.0022	0.6360	0.0465
	CPH-ISS	0.0030	0.0114	1.0000	0.8636	0.5032	0.0593	0.0267	1.0000	0.4799	0.6229	0.0245	0.0022	1.0000	0.0052	0.0416
	DDH	0.0070	0.0234	0.8636	1.0000	0.5901	0.4242	0.2544	0.4799	1.0000	0.9680	0.1327	0.6360	0.0052	1.0000	0.1003
	SCOPE	0.0231	0.0540	0.5032	0.5901	1.0000	0.5243	0.3707	0.6229	0.9680	1.0000	0.7586	0.0465	0.0416	0.1003	1.0000

Supplementary Table 15. p-values from pairwise t-tests with 4 $d.f.$ for difference of means between different methods in the OS prediction. We stratified the tests per study and per t_{cond}.

		AE-0				AE-1				AE-2				AE-3				AE-4				AE-5			
		RSF	CPH	DDH	SCOPE	RSF	CPH	DDH	SCOPE	RSF	CPH	DDH	SCOPE	RSF	CPH	DDH	SCOPE	RSF	CPH	DDH	SCOPE	RSF	CPH	DDH	SCOPE
$t_{\text{cond}} = 1$	RSF	1.0000	0.3855	0.1669	0.6794	1.0000	0.0999	0.0993	0.2569	1.0000	0.3884	0.3281	0.9592	1.0000	0.6283	0.1044	0.2299	1.0000	0.2751	0.7580	0.8043	1.0000	0.5625	0.5061	0.643
	CPH	0.3855	1.0000	0.0166	0.3105	0.0999	1.0000	0.8071	0.7303	0.3884	1.0000	0.8179	0.3190	0.6283	1.0000	0.0163	0.2958	0.2751	1.0000	0.3157	0.2550	0.5625	1.0000	0.8360	0.950
	DDH	0.1669	0.0166	1.0000	0.0359	0.0993	0.8071	1.0000	0.8735	0.3281	0.8179	1.0000	0.2678	0.1044	0.0163	1.0000	0.0219	0.7580	0.3157	1.0000	0.9186	0.5061	0.8360	1.0000	0.819
	SCOPE	0.6794	0.3105	0.0359	1.0000	0.2569	0.7303	0.8735	1.0000	0.9592	0.3190	0.2678	1.0000	0.2299	0.2958	0.0219	1.0000	0.8043	0.2550	0.9186	1.0000	0.6430	0.9500	0.8190	1.000

Supplementary Table 16. p-values from pairwise t-tests with 4 $d.f.$ for difference of means between different methods in the AE prediction on MM2 and for $t_{\text{cond}} = 1$. AE 0 to 5.

		AE-6				AE-7				AE-8				AE-9				AE-10			
		RSF	CPH	DDH	SCOPE	RSF	CPH	DDH	SCOPE	RSF	CPH	DDH	SCOPE	RSF	CPH	DDH	SCOPE	RSF	CPH	DDH	SCOPE
$t_{\text{cond}} = 1$	RSF	1.0000	0.2503	0.3422	0.0912	1.0000	0.2809	0.4344	0.3095	1.0000	0.6752	0.3175	0.1009	1.0000	0.5147	0.0870	0.2927	1.0000	0.1787	1.0000	0.6577
	CPH	0.2503	1.0000	0.1135	0.5623	0.2809	1.0000	0.9005	0.1102	0.6752	1.0000	0.2052	0.0562	0.5147	1.0000	0.1802	0.5374	0.1787	1.0000	0.1591	0.3394
	DDH	0.3422	0.1135	1.0000	0.0538	0.4344	0.9005	1.0000	0.1646	0.3175	0.2052	1.0000	0.6364	0.0870	0.1802	1.0000	0.5593	1.0000	0.1591	1.0000	0.6425
	SCOPE	0.0912	0.5623	0.0538	1.0000	0.3095	0.1102	0.1646	1.0000	0.1009	0.0562	0.6364	1.0000	0.2927	0.5374	0.5593	1.0000	0.6577	0.3394	0.6425	1.0000

Supplementary Table 17. p-values from pairwise t-tests with 4 $d.f.$ for difference of means between different methods in the AE prediction on MM2 and for $t_{\text{cond}} = 1$. AE 6 to 11.

	CPH-ISS vs. SCOPE				DDH vs. SCOPE			
	SS	d.f.	F	p	SS	d.f.	F	p
Method	0.060	1	77.290	$6.224e^{-12}$	0.021	1	19.298	$5.389e^{-05}$
Experiment	0.098	5	25.456	$5.430e^{-13}$	0.075	5	13.473	$1.745e^{-08}$
Residual	0.041	53	/	/	0.059	53	/	/

Supplementary Table 18. p-values from overall comparison between SCOPE, CPH-ISS, and DDH (with two-way ANOVA) on PFS prediction. SS stands for the sum of squares, d.f. stands for degrees of freedom, F is the F-statistic and p is the p-value.

	LOCF vs SCOPE				RNN vs SCOPE				DMM vs SCOPE			
	SS	d.f.	F	p	SS	d.f.	F	p	SS	d.f.	F	p
Method	1.063	1	33.426	$4.022e^{-07}$	0.044	1	145.787	$7.731e^{-17}$	2.933	1	1285.226	$7.699e^{-39}$
Experiment	3.324	5	20.912	$1.765e^{-11}$	0.355	5	233.602	$8.103e^{-35}$	0.632	5	55.400	$7.662e^{-20}$
Residual	1.685	53	/	/	0.016	53	/	/	0.121	53	/	/

Supplementary Table 19. p-values from overall comparison between SCOPE, LOCF, RNN, and DMM (with two-way ANOVA) on the forecasting task. SS stands for the sum of squares, d.f. stands for degrees of freedom, F is the F-statistic and p is the p-value.
